# Modern slavery and labor exploitation during the COVID-19 pandemic: a conceptual model

**DOI:** 10.1080/16549716.2022.2074784

**Published:** 2022-06-22

**Authors:** Tessa Washburn, Marissa L. Diener, David S. Curtis, Cheryl A. Wright

**Affiliations:** Department of Family and Consumer Studies, University of Utah, Salt Lake City, Utah, USA

**Keywords:** Human trafficking, public health, causal pathways, root causes, ecological systems theory

## Abstract

**Background:**

Modern slavery is a complex global health problem that includes forced labor exploitation. An ecological systems perspective is needed to understand how contextual upstream and midstream factors contribute to labor exploitation, and how disruptive societal challenges, such as infectious disease pandemics, may exacerbate established pathways leading to exploitation. Accumulation of familial and societal risk factors likely heightens vulnerability; for instance, economic precarity for an individual interacts with poor livelihood options and lack of social welfare supports increasing their likelihood of accepting exploitative labor. However, few frameworks exist that account for the accumulation of and interdependence between risk factors at different levels and across contexts.

**Objective:**

Using an ecological systems framework, we review literature on the pathways leading to labor exploitation, with the aim of developing a conceptual model grounded in existing research. Next, we discuss how pathways in this conceptual model are likely exacerbated by the COVID-19 pandemic. This conceptual model can guide future research to detect modifiable factors and strategic points of intervention.

**Methods:**

A critical review of research articles and gray literature was performed with a primary focus on sub-Saharan Africa. The review utilized various scholarly databases to identify perspectives from multiple disciplines and to more fully account for complex processes linked to labor exploitation.

**Results:**

A conceptual model of these pathways was developed that emphasizes established determinants and risk factors for labor exploitation in sub-Saharan Africa. The model highlights how the COVID-19 pandemic may have exacerbated these pathways.

**Conclusions:**

Future studies should carefully examine the direct and indirect pathways, accumulation of and interactions between factors, and specific external and personal stressors. Interdisciplinary research on multilevel interventions is needed to guide solutions to prevent the persistent problem of labor exploitation.

## Background

The eradication of modern slavery remains a pressing global health challenge. In 2016, approximately 40.3 million people globally were victims [1], with children younger than 18 years of age representing one third of all detected victims of trafficking [2]. Modern slavery is an umbrella term that refers to ‘exploitation that a person cannot refuse or leave because of threats, violence, coercion, deception, and/or abuse of power’ and includes the legal concepts of human trafficking, forced labor, and forced marriage [1]. The exploitative conditions and abuse experienced in forced labor, sex trafficking, and forced marriage engender severe mental and physical health consequences [3,4], and interfere with the development of human capabilities [5]. The impact of modern slavery likely reverberates through generations and perpetuates disadvantage [6,7]. The United Nations Sustainable Development Goals target modern slavery (namely, targets 5.2, 5.3, 8.7, 16.2 include eradicating forced labor and human trafficking and eliminating the worst forms of child labor [8]), yet little progress in attaining these goals has been made.

Development of effective solutions has been impeded by gaps in our understanding of the individualized paths leading to trafficking. These research gaps likely stem from the predominant focus on prosecution and law enforcement strategies with less emphasis on prevention [9,10]. Although social determinants and root causes of human trafficking have been described [2,11–17], limited research has documented how familial and societal risk factors accumulate across systems and heighten vulnerability [6,18,19]. These few studies have shown, for instance, that individuals who experience poverty, death or illness of a family member, and lack a social safety net have a greater likelihood of trafficking than individuals that confront only one of these risk factors [18,19]. Thus, a greater understanding of the ecosystem of factors (i.e. root causes and risk factors) that increase vulnerability and varied pathways to being trafficked is critical for effective public health prevention and intervention work [20,21]. Furthermore, major societal stressors, such as an economic crisis [2], disease outbreak [22], or climate change-related natural disasters [23], may exacerbate vulnerability to trafficking by increasing risk factors and disrupting existing sources of support. Significant research challenges exist given the complex phenomenon of modern slavery, including human trafficking’s clandestine nature, association with various economies [24], lack of reliable data and statistics [25,26], and fragmented information (e.g. sex trafficking receiving more attention than labor trafficking) [14].

### Aims

The aims of this paper are to develop a conceptual model depicting pathways leading to labor exploitation and to analyze the influence macroeconomic systems, institutions, and family factors can have. The focus is primarily on sub-Saharan Africa.

## Methodology

**Figure 1.**
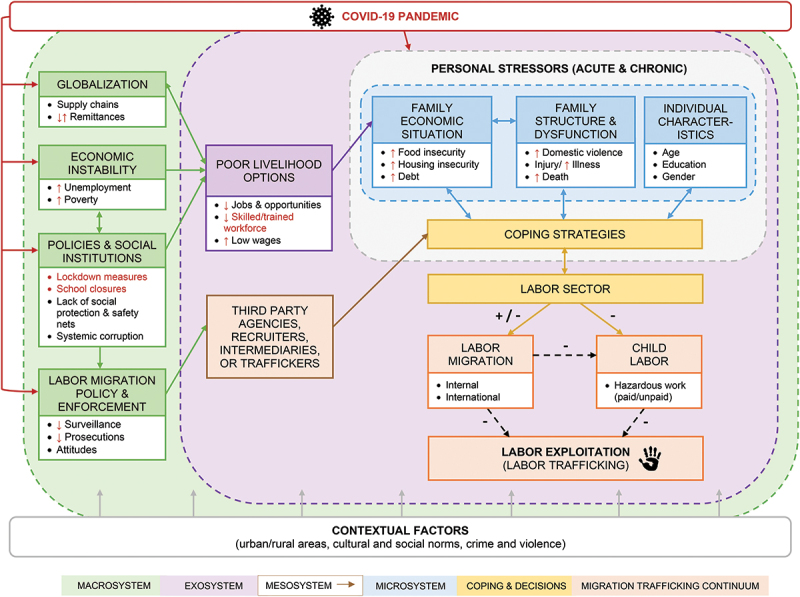
Conceptual model depicting multilevel pathways to labor exploitation and the potential influence of the COVID-19 pandemic.

Note. Some of the root causes and risk factors of labor exploitation are listed within each ecological system (i.e. macrosystem, exosystem, mesosystem, and microsystem). The dash lines represent permeability across ecological systems to show interconnections and how accumulation of risk factors may occur. The personal stressors (acute and chronic) box highlights contexts of persistent stress. Red text signifies the COVID-19 pandemic and how it might be intensifying risk factors and pathways. Individual characteristics and contextual factors are displayed which likely moderate the odds of labor exploitation.

To understand the pathways leading to labor exploitation and the influence of systems at different levels, we used Bronfenbrenner’s ecological systems theory [27]. Ecological systems theory provides a useful framework by highlighting the personal, interpersonal, institutional, and macro-level contexts in which human development and behavior occur. Bronfenbrenner’s ecological systems, macrosystem, exosystem, mesosystem, and microsystem, provided the framework for our critical review and conceptual model [27]. A critical review was conducted by synthesizing research from multiple disciplines relating to these four ecological systems and the influence of COVID-19 to develop a conceptual model [28] (see Figure 1). Specifically, we examined conditions in the macrosystem (i.e. laws, policies, and culture), risk factors in the exosystem and mesosystem (e.g. economic livelihood options, local institutions, and interplay between community groups and families), stressors experienced by families (i.e. microsystem), and the interactions between these systems. Within the literature reviewed, we also identified existing models that illustrated pathways to exploitation and trafficking and/or applied an ecological perspective to further inform our model [28]. Finding existing models is an essential step for conceptual model development [29]. Lastly, we reviewed articles on how the COVID-19 pandemic may have affected root causes and risk factors related to modern slavery, as well as organizational reports specifically about COVID-19’s known and likely effects on child labor and human trafficking.

### Materials

Our critical review collected data from multiple scholarly databases (e.g. EBSCOhost databases: Academic Search Ultimate, MEDLINE, CINAHL, and APA PsycInfo; Scopus (Elsevier); and Google Scholar). Initial searches included ‘human trafficking’ in combination with terms, such as ‘root causes,’ ‘causal pathways,’ and ‘social determinants.’ Developing the conceptual model was an iterative process: reading articles, developing an initial draft model, reading more articles, identifying existing models, revising the model, and integrating connections and gaps in extant literature into the conceptual model. Therefore, our search strategy and terms evolved through model development (e.g. ‘modern slavery,’ ‘COVID-19,’ ‘pandemic,’ ‘migration,’ ‘child lab*,’ ‘family’) and additional databases (e.g. PubMed and LitCovid) were searched. We identified gray literature in retrieved articles and conducted searches in Google with the terms mentioned above. Reports produced by organizations and agencies known to address modern slavery (e.g. International Labour Organization [ILO], International Organization for Migration, United Nations Children’s Fund [UNICEF], United Nations Office on Drugs and Crime [UNODC]) and economic development for poverty reduction (e.g. World Bank) were reviewed. Backward citation searching and forward citation searching in Google Scholar using keywords was performed. Two experts from different disciplines (economics and family-focused public health) also suggested articles. Literature retrieval primarily occurred from April 2020 to October 2021.

#### Migration and human trafficking continuum

Research suggests a strong link between human trafficking and labor migration [6,12,14,17–19,30–36]. Existing conceptual models of human trafficking have focused on this pathway [6,17,18,35–37], and some have included crises [6,17,37]. We expand upon these models [6,17,18,35,37] by exploring upstream and midstream factors that create vulnerability for labor exploitation and how an external stressor (COVID-19) may act as a catalyst. Since the majority of trafficking victims in sub-Saharan Africa (SSA) are in forced labor [2], and hazardous labor conditions more prevalent [38], we focus on this area. For this paper, a migrant is defined as ‘Any person who has moved – voluntarily or involuntarily – across an international border (international migrants) or domestically within a country away from their usual place of residence (internal migrants)’ [31]. Our principal emphasis is on voluntary migration [39,40]. Further, when referring to human trafficking, the widely used United Nations Trafficking in Persons Protocol (i.e. Palermo Protocol) definition is applied with exploitation being ‘sexual exploitation, forced labour or services, slavery or practices similar to slavery … ’ [41].

## Results

In this section, our conceptual model is described (Figure 1). The most common labor exploitation factors within each ecological system are explained, and then COVID-19 implications are discussed.

### Macrosystem and exosystem

#### Economic instability, poor livelihood options, and globalization

Poverty is frequently cited as a driving force that intensifies vulnerability to exploitation [2,17,18,31,42]. Lack of employment opportunities combines with poverty to increase willingness to accept exploitative work and labor conditions [12,18], influencing the supply side of trafficking [42]. Principally, globalization has magnified the push and pull factors that interact to facilitate migration and risk of labor exploitation. The push factors, which contribute to increasing out-migration, include poverty, unemployment, and unstable economies [43]. The pull factors that attract migrants to countries, include higher wages [44] and aspirations for better life opportunities [14]. Demand for low-cost labor is often driven by high-income countries (HICs), and the supply of laborers from low- and middle-income countries (LMICs) [42,44]. Further, advances in technology have increased access to information, global networking, and travel for migration [16,45]. As an example of these factors, in Ethiopia, the odds of migrants being trafficked were 1.49 times greater from rural areas, 2.55 times higher in the lowest household wealth quintile, and 8.64 times greater when migrants had strong feelings of hopelessness about the likelihood of achieving success in their home country [16]. While globalization provides opportunities for upward social mobility [6], it also expands the number of people at risk for labor exploitation.

#### COVID-19 implications

According to the World Bank, in 2020, 97 million new people were pushed into extreme poverty (<$1.90 per day) due to the COVID-19 pandemic [46]. Economic recovery is projected to be uneven with the global unemployment rate expected to be 5.7% in 2022, whereas in SSA it is estimated to be 6.4% [47]. COVID-19 has increased unemployment and underemployment resulting in reduced incomes [47]. A prolonged economic recovery will further affect those already hardest hit, such as women, youth, and informal workers [47]. In SSA, informal work is common (i.e. 85% of workers), with women and youth representing higher shares [47]. Underemployment in SSA has been intensified due to the pandemic (i.e. 7.1% decrease in work hours in 2020) [47]. In these ongoing distressed circumstances, acceptance of exploitative employment or questionable working conditions is more likely [2]. Migrant workers have also been affected by the termination of jobs, fewer social protection benefits, and disruptions to remittances [47]. In 2020, remittance flows to SSA saw a substantial decrease of 12.5% [48]. In the medium to long-term, labor migration and remittance flows will likely increase [49] due to quicker economic recoveries expected in HICs (pull factor) and slower recoveries in LMICs (push factor) [47].

#### Policies and social institutions, and labor migration policy and enforcement

Livelihood options are influenced by interactions between policies, weak social institutions, and the economic environment [12,13]. Economic instability and neoliberal policies instituted through structural adjustment programs (SAPs) have led to greater income inequality, economic decline, and poverty [12,50]. Implementation of SAPs with their austere macroeconomic policies has reduced social safety nets [43,43], resulting in fewer protections against financial instability for poorer households [50] potentially facilitating migration. Trafficking thrives in places of systemic corruption, crime [14], feeble enforcement of laws, and inadequate prosecution of traffickers [17]. Thus, a higher prevalence of crime combined with poorer livelihood options could push individuals to migrate [6]. However, a single focus on prosecution creates an oversimplification of trafficking, restrictive immigration policies, and justification for strict border control measures [10,51]. While human trafficking is a global and lucrative business [33,42], involving high profit with low risk [13,15], the amount of money is dependent upon the type of exploitation [2].

#### COVID-19 implications

Policies enacted in response to the COVID-19 pandemic, or the lack thereof, have unintentionally magnified some of the pre-existing conditions that increase vulnerability to labor exploitation. However, some countries in SSA have extended temporary relief measures to poor households and enterprises during the pandemic [52]. In 2020, approximately 250 million children in SSA were affected by school closures raising concerns that millions would never return [53]. School closures intensify the risk of child labor [54] and can have short and long-term effects on human capital [55]. Prolonged disruptions to education and skill-based training affects opportunities for higher wage employment among youth [47], likely leading to lower wages and informal work.

Understaffed or overwhelmed law enforcement agencies assisting with the response to the COVID-19 pandemic may result in resources and attention being diverted away from investigating human trafficking [56–58]. In a survey conducted by UNODC [56], respondents from 46 countries disclosed challenges they encountered with law enforcement activities during the COVID-19 pandemic. Common law enforcement challenges were 44% indicating fewer public reports about suspected cases of trafficking, 33% describing difficulties with collecting information for investigations due to lockdown measures, and 24% revealing no labor inspections were done [56]. These factors, among others, have resulted in postponed investigations and prosecutions in conjunction with closed courts or suspended hearings [56].

### Mesosystem

#### Third party agencies, recruiters, intermediaries, or traffickers

Migrant networks and intermediaries assist with job placement, whether exploitative or good working conditions [6,31]. Traffickers include organized criminal groups, opportunistic groups, or individuals [2], with former victims of trafficking also represented [43]. In SSA, the majority of traffickers are men, but in West Africa, there is a greater prevalence of women who are traffickers [2]. The early interactions between agents, recruiters, or traffickers with individuals or families remains complex. In Nigeria, some individuals or families will seek an agent to assist them with finding work and/or their migration journey [12]. Placing children with wealthier families or friends is a cultural tradition in SSA, but this practice can also lead to exploitative labor [12,15,59]. Other times, a recruiter, such as someone well known in the community, family member, or friend, will approach families [12,15]. An existing relationship and knowledge of the individual or family’s vulnerable circumstances likely makes deception easier, particularly during times of distress [18].

#### COVID-19 implications

As the COVID-19 pandemic has expanded the need for labor migration due to declining employment opportunities, unscrupulous recruiters or traffickers will likely, either in person or through the internet, leverage existing or new relationships to deceive distressed individuals. Others will wait for people to respond to attractive employment opportunities or assistance with migration [2]. With decreases in prosecutions, labor inspections, and unstable economic conditions, UNODC indicates traffickers have capitalized on the situation and devised new schemes for exploitation [56].

### Microsystem and personal stressors (acute and chronic)

#### Family economic situation, and family structure and dysfunction

In UNODC’s analysis of 233 court cases, economic need was the principal risk factor that traffickers exploited (51% of court cases) [2]. This corroborates other research that suggests when an individual or a family’s economic situation declines, desperation may increase vulnerability [11,18,43] to deceptive financial solutions [2]. Qualitative interviews, with key informants and trafficking survivors in Nigeria, revealed that employment decisions were influenced by perceptions of an individual’s role within their family [19]. The second most common risk factor identified by UNODC was living in a dysfunctional family (20% of court cases) [2]. Vulnerability to labor exploitation might increase when an individual has experienced prior abuse, especially for children [60]. Perhaps perpetuated by financial distress, domestic violence and abuse could propel migration and acceptance of attractive work opportunities [11]. Financial strain and family dysfunction can even result in family members exploiting other family members [2]. In a sample of about 12,000 survivors of child trafficking, approximately 41% of the cases involved a family member or relative, either intentionally or unintentionally, during the initial phases [61].

#### COVID-19 implications

Phone surveys conducted with a representative sample of over 30,000 households from nine different countries, with five from SSA (i.e. Ghana, Rwanda, Sierra Leone, Kenya, and Burkina Faso), found decreases in food security, income, and employment (median share 45%, 70%, and 30%, respectively) during the pandemic [62]. Moreover, families are directly affected when an economic earner dies from COVID-19 or suffers long-term illness impeding their ability to work likely resulting in other family members working to support the family. Nearly 20% of youth aged 15–17 years in SSA engage in hazardous work, whereas the next highest region is 9.8% [63]. An increasing youth bulge in SSA [64]; higher levels of unemployment, underemployment, and poverty due to COVID-19 [47]; and perceptions of individual roles within family may escalate the acceptance of precarious employment among youth in the informal sector. Additionally, reports of domestic violence have risen during the pandemic [65].

#### Individual characteristics

Several individual characteristics have been found within the labor migration and trafficking literature that might moderate the likelihood of labor exploitation. We highlight three significant characteristics: age, gender, and education. Age has been identified as a risk factor [2,16,31], but this varies by geographical area. Of detected victims of trafficking, more than half in SSA (59%) are children (<18 years of age) [2]. How age may be a risk factor for labor exploitation in SSA is explored further in the section about child labor. Gender has been extensively cited in the literature as a key determinant [2,11,31]. However, the type of labor sector matters for which gender is most at risk for exploitation (see coping and decisions section) [2]. Higher levels of formal education appear to be protective [17] as lower education levels are associated with exploitation [11,16,30]. Yet, the extent of this relationship remains unclear [31]. The Counter Trafficking Data Collaborative [66] indicates that more than half of the identified cases had technical training or gone to middle school.

### Coping and decisions

#### Coping strategies

Resiliency to one stressor could wane as other stressors emerge or circumstances evolve [67]. The Family Adjustment and Adaptation Response (FAAR) Model postulates that families balance demands (daily hassles, stressors, and prolonged family strains) with their capabilities (resources and coping behaviors) and family meanings (perceptions of stressors and capabilities) [67,68]. For many families, the COVID-19 pandemic presents new demands on top of existing ones that further challenge their capabilities. The family’s level of distress [69], available resources, and shared family meanings [67] will influence their selection of coping strategies, such as skipping meals [62], selling assets, and using savings [70].

Migration is an adaptation [71] and family strategy [72]. For example, the new economics of labor migration theory proposes that poor households will manage risks through migration to receive remittances while other family members labor locally [73]. Migration decision-making is a complex phenomenon influenced by the interplay of individual characteristics, social and structural factors, and knowledge [74]. Migration success stories seem to be influential [15,45]. Greater risk of exploitation could occur for uninformed individuals [17]; however, some argue most migrants are aware of the possible dangers but move internally or internationally because of their circumstances [19]. Limited knowledge of migration processes could increase vulnerability, as migrants may have to rely on fragmented sources of information or networks [31]. Zimmerman and Kiss [6] have suggested that urgent migration decisions ignited by conflicts or crises put individuals at greater risk of exploitation. Therefore, understanding household decision-making regarding migration and risk management strategies is essential and likely involves multiple household members [75]. Additionally, prior encounters with agents or traffickers within the community could influence decisions about labor migration domestically or internationally.

#### Labor sector

Who migrates (e.g. family, caregiver, or child/children) is influenced by earning potential and the types of opportunities available [72], cultural and social norms [76], individual characteristics, and social networks. In some contexts, devaluation of women and girls [13,17] and gender division of labor [44] could contribute to decisions. With the demand for low-wage female labor [32], women account for almost half of all international migrants [77] and might find work easier than men [44]. How the COVID-19 economic crisis will affect these pathways remains uncertain, as women have experienced considerable job loss and increases in unpaid activities [47]. Economic labor sectors also affect decisions of who migrates. Whether the migration is internal or international, specific industries are associated with labor exploitation [1]. Domestic work, especially when it leads to domestic servitude, places girls and women in vulnerable positions of abuse, limited freedoms, and delayed or unpaid wages [1,2]. Other sectors with higher incidences of labor exploitation involve arduous manual labor, including agriculture, fishing, construction, and manufacturing, which typically involve boys and men [1,2].

### Labor migration

It is imperative to mention that because someone migrates does not mean they will become victims of labor exploitation [31], though, some will encounter diverse forms of syndemic vulnerability [78]. Zimmerman and Kiss [6] have stated, ‘While migration within and across national borders has been an economic and social mobility strategy that has benefited millions of people around the world, … labor exploitation of migrant workers has become a problem of global proportions.’ Some forms of migration increase vulnerability, such as displacement [79]; informal employment [14]; illegal or irregular migration [6,16], including using irregular migration channels [31] or routes [19]; crossing borders [31]; and international [1] where language is a major barrier [18]. Furthermore, exploitation increases when the specific labor sector isolates migrants and limited protections from the State are in place [31].

### Child labor

Countries with a higher prevalence of child labor also have more children detected as victims of trafficking [2]. This association is stronger in low-income countries where child trafficking predominately occurs through forced labor [2]. The ILO Conventions 138 [80] and 182 [81] and Recommendation 190 [82] specify the differences between light work, child labor, hazardous work, and the worst forms of child labor (WFCL). Child labor is when children less than 12 years old are participating in any type of economic activity, children 12–14 or 13–15 years old doing more than what is defined as light work [80], and 15–17 years old engaged in hazardous work [83]. Hazardous work is when children labor in dangerous environments, such as operating machinery, working long hours or at night, exposure to substances, or any kind of abuse [82]. For anyone less than 18 years old, the WFCL is all forms and related practices of slavery, including child trafficking, forced labor, and any work that harms the morals, safety, or health of children [63,81].

In places where child labor is common and culturally accepted, traffickers can exploit children for labor more easily [2]. Therefore, child labor might be an alternative pathway to exploitation. Although child labor and trafficking are different, they share similar risk factors, including economic instability and poverty [83,84]; exposure to or accumulation of shocks/stressors [63,85]; urban/rural disparities and poor livelihood options [86]; death, illness, or injury to a primary economic earner [85]; and gender inequality [63,86]. Other important factors, for child labor, are socialization [87], accessible and quality schools [63,85,86], and access to credit [86]. Child labor is often used as a coping mechanism while families confront stressors, especially when there are inadequate social protection systems in place [85]. If social protection efforts remain the same or worsen due to the implementation of austere economic policies in response to the COVID-19 economic crisis, a model predicts that between 8.9 to 46.2 million more children will be in child labor by the end of 2022 [63]. Seasonal, irregular family migration, or independent child migration increases the risk of child labor [88]. In LMICs, independent child migration generally occurs internally [88], and the likelihood of child labor is due to existing risk factors and how the migration occurs [89].

## Discussion

For greater prevention of labor trafficking, the conditions and modifiable factors that increase vulnerability of exploitative labor need to be targeted [9]. Our conceptual model was based on previous models and research, especially the work by Zimmerman and Kiss [6], with a focus on the accumulation of risk factors [6,18,19] across ecological systems. It is not comprehensive of all the contextual factors or potential pathways leading to labor exploitation. Rather, we describe driving macroeconomic forces and the role of local institutions in creating the conditions that sustain a market for exploitation, and how family stressors arise within these circumstances to increase vulnerability. Although there are limitations with critical reviews which use non-systematic search methods, such as researcher subjectivity and non-replicability of review procedures, our purpose was to provide a multidisciplinary conceptual model on a timely public health problem. A critical review was done to guide future research [28] and identify potential intervention points [29] by investigating how individual vulnerability to exploitative labor is embedded in institutions and systems, with risk accumulating across systems. Future studies need to be multilevel and include assessments of relevant systems and interdependencies between them (e.g. interactions between local institution- and family-level risk factors, direct and indirect pathways, and how systems and pathways are affected by crises). Adaptation of the conceptual model will be essential as the system-level factors and pathways are likely to vary by country, political systems, and stressors. Thus, how the COVID-19 pandemic is augmenting existing systems and risk factors is likely to vary in key ways from other stressors.

### Recommendations and future directions

While the COVID-19 pandemic presents numerous challenges, it also provides opportunities to develop safer migration policies, more resilient supply chains, extend social protection to the most marginalized, and promote sustainable human development globally [90,91]. However, the path forward remains unclear, especially as human trafficking, labor exploitation, and labor migration in particular, remains a politicized and contested topic. Applying the 4Ps human trafficking paradigm (i.e. prevention, protection, prosecution, and partnership) [92], we provide some recommendations for future research and practice relating to the ecological systems described in this paper.

#### Macrosystem and mesosystem

In the conceptual model, we illustrate how *prosecution* is connected to macro-level migration enforcement policies and traffickers in the mesosystem. Gaps remain with labor migration policies and law enforcement strategies [9,51], and focusing only on arrests will not eliminate exploitation [21]. Restrictive immigration policies and border controls, intentionally or unintentionally, limit who can migrate safely and legally, driving unsafe migration while simultaneously overlooking structural factors and the role of nation states – implementing policies that perpetuate globalized capitalism [10,51,93]. Our model highlights the role of globalization and how HICs profit off cheap labor and LMICs benefit from remittances (see macrosystem and exosystem section) [94]. However, the varied political and ideological dynamics associated with trafficking and migration are only partially illustrated in the model (e.g. ‘labor migration policy and enforcement’ and ‘attitudes’).

A thorough analysis to identify gaps in current migration *protection* policies and their implementation should be conducted, focusing on where and what kinds of expansion for legal migration could be made to overcome harmful policies [31] that currently control rather than *protect* migrants [51]. Additionally, more people may move internally when stricter COVID-19 travel and border measures are in place. Therefore, understanding the risks internal migrants encounter is needed [1], especially since most migration occurs internally [95]. Approximately 58% of human trafficking victims are detected domestically [96], yet the literature focuses on international migration. Consequently, studies on international migration provide much of the empirical support for our conceptual model, and some of the system-level factors and pathways for internal labor migration and exploitation might vary. Urgent investigation is also needed to understand how the pandemic has affected complex supply chains [91] in relation to labor exploitation [33,97].

Significant policy challenges remain due to broadly defined concepts and legal terms, various interpretations of the Palermo Protocol, disagreements about approaches, and whether exploitation creep by conflating forced labor, human trafficking, and slavery terms impedes or facilitates reduction in trafficking [10], all of which lead to imprecise prevalence estimates [25,26] and uncertainty whether progress is being made. Although we see some overlap in the pathways leading to different forms of modern slavery and use these terms interchangeably, agreeing on legal definitions and corresponding surveillance and measurement approaches is vital to support research on effective policies, and to facilitate a greater understanding of the prevalence of labor exploitation and other forms of modern slavery. Nevertheless, clarifying definitions and improving surveillance will not address the root causes and structural systems driving exploitation on a global scale. *Partnerships* that include interdisciplinary and multisector collaboration and implementation of multilevel evidence-based interventions are needed to achieve progress in addressing the root causes of labor exploitation [98].

#### Exosystem

Asset-based approaches should be considered [19,99,100] to improve livelihood options for those living in poverty. External funds must be used judiciously to invest in individuals, families, and communities and to bolster community-identified deficits as resources are further constrained due to COVID-19. Utilizing strength-based or asset-based approaches when addressing the accumulation of risk factors is vital to fostering stronger community *partnerships* to illuminate contextual factors and tailored solutions to assist with *prevention*. In other words, applying a deficit-based focus could severely impede *partnerships* and *prevention* efforts. While philanthrocapitalism has potential to build on individual and community strengths, attentive evaluation before and continuously throughout must occur to *prevent* harmful and unintentional effects of capitalism that perpetuate labor exploitation [10,94]. Increasing social protection in the macrosystem to help those in economic need in the microsystem is recommended for reducing both child labor [63,85] and trafficking [2], and may buffer against poor livelihood options. Expanding social protection to families through cash transfers has been found to decrease participation in child labor [101], reduce short-term poverty [102], improve school enrollment, but mixed results with school attendance [103]. The effect size of cash transfers are subject to context, program design [85], and whether other components are incorporated [100,103].

#### Microsystem

Research on the *prevention* of human trafficking is scarce [20], especially when focusing on families. A greater understanding of family dynamics and decision-making in child labor research is also needed as over two-thirds of children (72% globally, 82% SSA) that are engaged in child labor work in family enterprises or family farms [63]. Future research should explore different family dynamics, decision-making, and how to link protective factors across ecological systems [67] for comprehensive multi-component interventions [100]. For COVID-19 specifically, our model highlights how job loss or reduced wages together with school closures might intensify child labor decisions. Research should investigate whether reduced wages and school fees affected enrollment when schools reopened after lockdowns [54]. In particular, this research should focus on 15–17 year-olds, who are above the minimum working age, but are more likely to engage in hazardous work [83]. Both remedial action and innovative approaches to reach children simultaneously working and going to school or exclusively working are needed with the COVID-19 school closures [55]. For example, approaches could include evening schools [104] or marketable skill-based training programs [17]. These efforts should complement community-based interventions [2] to improve livelihood options and strengthen financial security for vulnerable families.

Understanding the differences in phenomena between child labor and child labor trafficking related to migration is critical for interventions [105]. Preliminary research suggests that local child laborers work in better conditions than migrant child laborers, but this should be investigated further [89]. For additional prevalence estimates of child labor and child labor migration, household surveys, such as UNICEF’s Multiple Indicator Cluster Surveys [106], which have a child labor module, or school-based surveys could include supplementary questions about siblings’ labor activities and if any have migrated for work. Hazardous work is sometimes used as a proxy for the WFCL [63], but additional methodological research is required [107]. Furthermore, measures to detect conditions of hazardous unpaid household services are needed to complement current measurements that assess number of hours [108,109].

Given how context dependent exploitation is within communities [99], applying a positive deviant approach [110] to discover viable protective factors, mechanisms vulnerable families are using to thrive, and learning from individuals who have migrated safely, even with more risk factors, could prove invaluable [111]. Maternal education may protect against exploitation [17], but this relationship needs to be examined further and whether protective in SSA. Although challenges exist with statistics, researchers should consider using data from the Counter Trafficking Data Collaborative [66] to conduct various analyses. Finally, these suggestions are not exhaustive, beneficial recommendations are found in these articles [9,19,20,31].

With any of these approaches or others, evaluation is essential to public health work, and ascertaining fidelity, effectiveness, and any unintentional effects of programs. Few robust evaluations have been conducted on anti-trafficking campaigns [112] and *prevention* efforts [113,114], and this is a significant gap in human trafficking research [20,115]. Considerable challenges remain with *prevention* efforts to reduce labor exploitation. Evaluation and sharing what is working well and not is vital to addressing this perplexing global health issue [112,116].

## Conclusion

There is an urgent need for researchers and practitioners to understand the different multilevel pathways and modifiable factors that lead to labor exploitation in distinct contexts. Our ecological framework model specifies root causes that are crucial to address for effective prevention efforts. If policies and institutions fail to address these root causes, social determinants of health, and to promote human development, then marginalized populations facing economic precarity will continue to suffer from vulnerability to exploitative labor, especially during periods of crisis. Furthermore, multisector collaboration and multilevel interventions are essential for prevention. Until these changes occur, the pernicious and unjust problem of labor exploitation will persist and continue to flourish.

## Paper context

Root causes of human trafficking have been described, but less is known about how risk factors accumulate across ecological systems and are shaped by global and societal stressors. This review examines likely pathways to labor exploitation, the enabling social systems and institutions, and how the COVID-19 pandemic is exacerbating risks across systems. Interdisciplinary collaboration and multilevel interventions are required to disrupt these pathways and the interconnected risk factors to prevent labor exploitation globally.
